# Antimicrobial and anti-biofilm activity of hexadentated macrocyclic complex of copper (II) derived from thiosemicarbazide against *Staphylococcus aureus*

**DOI:** 10.1038/s41598-018-26483-5

**Published:** 2018-05-23

**Authors:** Umarani Brahma, Richa Kothari, Paresh Sharma, Vasundhra Bhandari

**Affiliations:** 1 National Institute of Animal Biotechnology, Hyderabad, Telangana India; 2 ITM University, Gwalior, Madhya Pradesh India

## Abstract

Multidrug-resistant pathogens causing nosocomial and community acquired infections delineate a significant threat to public health. It had urged to identify new antimicrobials and thus, generated interest in studying macrocyclic metal complex, which has been studied in the past for their antimicrobial activity. Hence, in the present study, we have evaluated the antimicrobial activity of the hexadentated macrocyclic complex of copper (II) (Cu Complex) derived from thiosemicarbazide against Gram-positive and Gram-negative bacteria. We observed increased susceptibility against standard isolates of *Staphylococcus aureus* with a minimum inhibitory concentration (MIC) range of 6.25 to 12.5 μg/mL. Similar activity was also observed towards methicillin resistant and sensitive clinical isolates of *S. aureus* from human (n = 20) and animal (n = 20) infections. The compound has rapid bactericidal activity, and we did not observe any resistant mutant of *S. aureus*. The compound also exhibited antibiofilm activity and was able to disrupt pre-formed biofilms. Cu complex showed increased susceptibility towards intracellular *S. aureus* and was able to reduce more than 95% of the bacterial load at 10 μg/mL. Overall, our results suggest that Cu complex with its potent anti-microbial and anti-biofilm activity can be used to treat MRSA infections and evaluated further clinically.

## Introduction

Antimicrobial resistance (AMR) has emerged as a global threat to public health and have become the leading cause of mortality. *Staphylococcus aureus* is one of the primary cause of nosocomial infections associated with increased morbidity^1,2^. Methicillin-resistant *S. aureus* (MRSA) is solely responsible for many life-threatening nosocomial infections in humans which increases the treatment duration and medical costs^3,4^. They are also known to cause several mild to chronic infection in animals. Resistance against the last resort of drugs such as vancomycin and linezolid have emerged in the clinical isolates of *S. aureus* which have further worsened the scenario^5,6^. Further, the problem of resistance is impended by the ability of *S. aureus* to form biofilms on biotic and abiotic surfaces especially on several medical implanted devices^7–9^. Biofilm forming *S. aureus* is even more significant threat due to increased tolerance against antimicrobials and the host defence system^3,9^. Biofilms are challenging to treat and considered as a bacterial reservoir for dissemination to various body sites^8,10^. These infections may result in the replacement of the implanted medical device, thereby causing increased distress with additional medical costs to the patient^10–12^.

With the increasing mortality rate due to AMR, there is an urgent need to look for new strategies to develop antibiotics to fight multidrug-resistant and biofilms related infections^13^. Researchers are using various approaches to identify new antibiotics or new compounds with antimicrobial activity. Various inhibitors of different pathways, antimicrobial peptides derived from different ecosystems, and organic and inorganic synthetic compounds with antimicrobial activity have been identified and studied^14–22^. In quest of finding new compounds with antimicrobial activity along with the scarcity of new antibiotics in the pipeline, have drawn the focus towards development of Copper (Cu) complexes as antimicrobial^18–22^.

Cu is one of the most investigated metal ions among other transition metals and hold importance due to their significant role in the various biological activity^22^. During intracellular bacterial infections, macrophages expose bacteria to increased Cu concentrations resulting in phagosomal killing^23^. Various reports of Cu complexes showing antibacterial and anticancer activity have been published earlier^21,22^. The hexadentated macrocyclic complex of copper (II) derived from thiosemicarbazide has shown potent antibacterial activity, which encouraged us to investigate its therapeutic potential^22^. The primary aim of our study is to determine the antibacterial activity of the Cu complex against clinically important bacterial pathogens and to investigate their mechanism of action, antibacterial and antibiofilm efficacy and capacity to kill intracellular bacteria.

## Methods

### Bacterial cultures

We used standard isolates of *Enterococcus faecalis, Staphylococcus aureus, Pseudomonas aeruginosa, Acinetobacter baumanii, Clostridium difficile, Klebsiella pneumonia, and Escherichia coli in* the study (Table 1). Antimicrobial susceptibility was determined in *S. aureus* clinical isolates from animal (n = 20) and human (n = 20) infections.

**Table 1:** MIC values against Gram-positive and Gram-negative bacteria.

Organism name and Strain ID	MIC Cu Complex (μg/mL)
*Staphylococcus aureus* (MSSA), ATCC 29213	6.25
*Staphylococcus aureus* (MRSA), ATCC 33592	6.25
*Staphylococcus aureus* (MRSA), ATCC 700699	12.5
*Enterococcus feacalis*, ATCC 29212	>100
*Escherichia coli*, ATCC 25922	>100
*Pseudomonas aeruginosa*, ATCC 27853	>100
*Klebseilla pneumoniae*, ATCC 700603	>100
*Acinetobacter baumanii*, ATCC BAA-747	>100
*Clostridium difficile*, ATCC 9689	>100

### Synthesis of Cu complex

**Figure 1:**
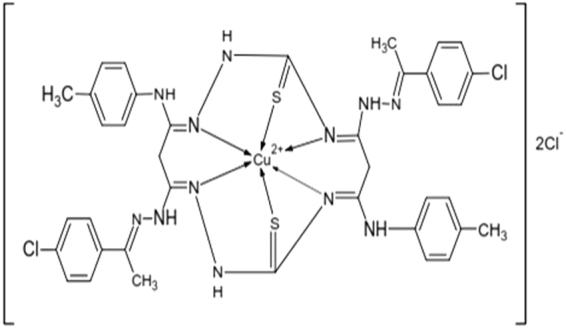
Structure of Macrocyclic Copper Complex.

The Cu complex was synthesised by the condensation reaction between substituted carbohydrazone and thiosemicarbazide in the presence of Copper chloride as described earlier^22^. Briefly, a divalent Copper chloride (1 mmol) solution was added to a stirred hot methanolic solution (≈50 cm^3^) of thiosemicarbazide (2 mmol) and substituted carbohydrazone. Further, the solution was refluxed for 8–10 h. The mixture was concentrated and kept overnight in desiccators. After overnight cooling, it formed a greenish coloured precipitate which was filtered, washed with methanol and dried in vacuum (Fig. 1). The compound obtained was assessed for its physical and analytical properties as described previously^22^.

### Antimicrobial susceptibility

Micro-broth dilution assay determined the minimum inhibitory concentrations (MICs) in 96 well plate format. The micro-broth dilution assay was performed as per guidelines of Clinical and Laboratory Standards Institute (CLSI) with a slight modification by using resazurin dye as described earlier^24^.

### Time-dependent Killing

ATCC 29213 (10^6^ CFU/mL) was inoculated in Mueller Hinton Broth (MHB) containing Cu complex dilutions (0.5 × MIC, 1 × MIC, & 2 × MIC) respectively in a final volume of 100 mL, were incubated in orbital shaker at 37 °C for 10 h^25,26^. The growth was monitored by taking absorbance at 600 nm for each drug concentration at various time points (0, 2, 4, 8, & 10 h) and compared with control culture with no drug. At each time points, an aliquot of 200 μl was serially diluted and plated on TSB agar for enumeration of colony forming units (CFU).

### Resistance Detection Study

*S. aureus* (ATCC 29213 and ATCC 700699) cells were adjusted to the count of 10^10^ CFU/mL and were plated on Mueller Hinton agar (MHA) plates containing 2×, 4× and 10× of the MICs value. The plates were then incubated for 48 h at 37 °C and observed for growth^27^.

Serial passaging of the bacteria in the sub-inhibitory concentration of drug was done to evaluate the development of resistance as described earlier^1,27^. Briefly, MIC values of ATCC 29213 strain against Cu complex and ofloxacin were determined by micro-broth dilution assay. The bacterial cells growing at a sub-inhibitory concentration (0.5 × MIC) of the compounds (Cu complex and ofloxacin) were harvested and inoculated into fresh media. The inoculated bacterial cells were subjected to another MIC assay. After 18–24 h incubation, cells growing in the second highest concentration from the previous passage were again inoculated and used for the MIC determination assay. We repeated the procedure for 15 passages. The fold change in MIC was plotted against the number of passages. The experiment was performed in triplicates.

### Anti-biofilm activity

The inhibitory effect of Cu complex on biofilm formation of *S. aureus* isolate was determined by 96-well plate-based Crystal violet (CV) assay^1,28^. An overnight culture of *S. aureus* (ATCC 33592) was diluted 1:200 in Tryptic Soy Broth (TSB, containing 0.25% glucose and 0.5% NaCl) and dispensed into 96-well plate (200 µL/well). The plate was incubated at 37 °C for 24 h and washed gently three times with PBS to remove planktonic bacteria. After washing, Cu complex and vancomycin was added ranging from 0 to 125 μg/mL, and the plate was incubated for another 16 h at 37 °C. All the wells were then again washed with PBS three times and fixed with methanol for 15 minutes. The plate was air dried for 30 minutes and 0.1% CV solution was added to each well and incubated at room temperature for 20 minutes. The picture of the wells was taken using a digital camera. After washing with distilled water, 33% acetic acid was added to each well and absorbance was taken at 590 nm. Mean absorbance values of each sample was calculated and compared with the mean values of controls.

Similarly, the anti-biofilm activity was also assessed using resazurin. The protocol used was same as mentioned above with slight modifications^29,30^. Briefly, the anti-biofilm effect of Cu complex was determined on 24 h and 72 h old biofilms. The pre-formed biofilms (24 h and 72 h) were treated for 24 h with different dilutions of Cu complex (0 to 125 μg/ml). After treatment, the wells were washed with PBS, and 100 μL of resazurin was added to each well and incubated for 30 min at 37 °C. The fluorescence was measured by using a multimode reader (Perkin Elmer, excitation wavelength = 550 nm and emission wavelength = 590 nm). The results were expressed as percent cell viability in treated wells as compared with untreated control. All experiments were repeated thrice in quadruplicates.

### Determination of the anti-biofilm activity by confocal microscopy

An overnight grown bacterial culture (diluted 1:200) was added to the chambered slide and incubated at 37 °C for 24 h^1^. The planktonic cells were carefully removed and washed with 1 × PBS. The Cu complex (50 µg/mL) was added to the slide and incubated for 4 h. Wells containing only medium were treated as control. After incubation, the wells were washed with 1 × PBS and stained with SYTO-9 (3 µM) and Propidium Iodide (PI, 15 µM) for 20 minutes and images were taken using a confocal microscope.

### Cell membrane permeability

A cell membrane permeability assay was performed using propidium iodide (PI). The PI uptake was determined by flow cytometry (FACs). Bacterial cells of *S. aureus* (ATCC 29213) was grown to exponential phase in TSB and incubated with 50 μg/mL of Cu Complex at 37 °C for 60 min. PI was added to the treated cells (10^6^ CFU/mL) at final concentrations of 5 μM. The cells were incubated in the dark at 25 °C for 5 min, and the sample was run on a flow cytometer. 10,000 events were recorded for control and treated samples.

### Scanning Electron Microscopy

ATCC 29213 cells were grown till the exponential phase and resuspended at a concentration of 10^8^ CFU/mL^31^. Cells were incubated with Cu Complex (50 µg/mL) for 60 min at 37  °C and cells without drug were used as a control. Further, the cells were pelleted and fixed with 2% glutaraldehyde solution for 2 h at 4 °C as described earlier^31^. The cells were dehydrated in a graded series of alcohols. The samples were then coated by sputter coater and were observed with Zeiss ULTRA 55 scanning electron microscope (SEM).

### Cell Cytotoxicity Assay

Cell cytotoxicity was determined using mouse macrophage adherent cell line, RAW 264.7. 5 × 10^3^ cells were seeded per well in 96 well tissue culture plate and allowed to adhere in DMEM medium containing 10% FBS for 24 h at 37 °C, 5% CO_2_. Cu Complex was added at different concentration (0, 1, 10, 50 & 100 μg/mL) in the cells in DMEM medium containing 10% FBS. After 24 h of incubation, resazurin dye was added to each well and incubated for 6–8 h at 37 °C, 5% CO_2_. The fluorescence was measured (excitation wavelength, 550 nm; emission wavelength, 590 nm) using the multimode reader. The results were expressed as percent cell viability, compared with untreated cells.

### Intracellular activity of Cu complex

RAW cell line 264.7 was seeded at a density of 50,000 cells per well in a 24 well tissue culture plate at 37 °C, 5% CO_2_. Macrophage cells were then infected with ATCC 29213 for 2 h at a 1:10 multiplicity of infection (macrophage to bacteria ratio). Cells were then washed with 1 × PBS twice, and gentamycin 50 μg/mL was added to each well to kill extracellular bacteria for 1 h. Cells were washed with 1 × PBS and incubated again for 24 h with different concentrations (0, 0.1 μg/mL, 1 μg/mL, 5 μg/mL, 10 μg/mL) of each antibiotic (oxacillin, vancomycin, linezolid and Cu Complex) at 37 °C, 5% CO_2_. After 24 h, the cells were washed twice with 1 × PBS and lysed using 0.1% saponin. The cell lysates were diluted and plated on tryptic soy agar plates, and colony-forming units (CFU) was counted. The results were expressed as % bacterial survival at different drug concentrations in comparison to control cells.

## Results

### Antimicrobial Susceptibility Profile of Cu complex

**Figure 2:**
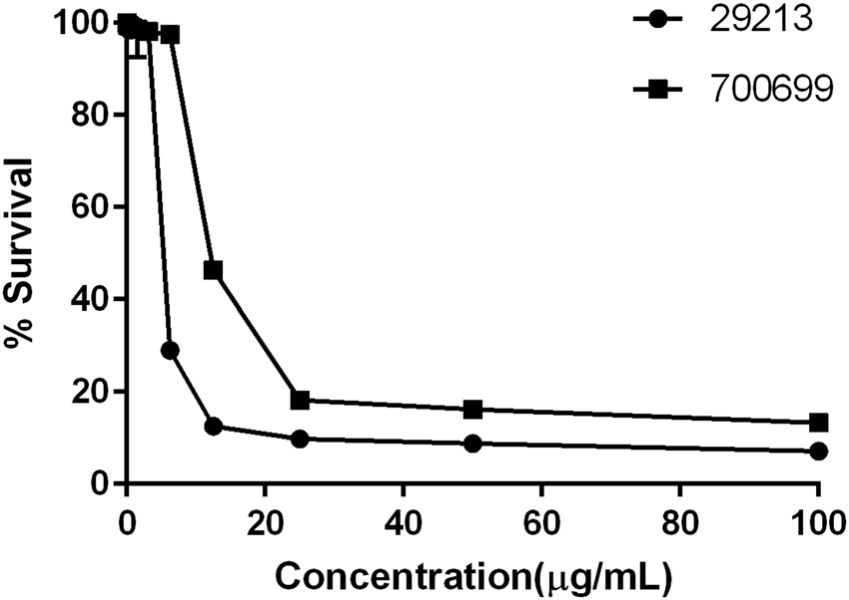
Antimicrobial Susceptibility profile of Standard *Staphylococcus aureus* isolates against Cu Complex. ATCC 29213 (sensitive) and ATCC 700699 (resistant isolate) susceptibility profile by micro-broth dilution assay. The graph represents mean % survival at different concentrations of Cu complex using resazurin assay.

Antibacterial activity of the Cu complex was evaluated against Gram-negative (*Pseudomonas aeruginosa, Acinetobacter baumanii, Klebsiella pneumonia*, and *Escherichia coli*) and Gram-positive (*Staphylococcus aureus, Clostridium difficile and Enterococcus faecalis*) bacteria (Table 1). Antimicrobial activity was observed against methicillin resistant and sensitive (MRSA & MSSA) isolates of *S. aureus*, which showed MICs ranged from 6.25 to 12.5 μg/mL) (Fig. 2). Further, reduced susceptibility with a MIC of 50 μg/mL was seen against *E. faecalis*. However, no antibacterial activity against Gram-negative bacteria was found up to 100 μg/mL.

### Susceptibility profile of Clinical isolates of *S. aureus* and no detectable resistance against Cu complex

**Figure 3:**
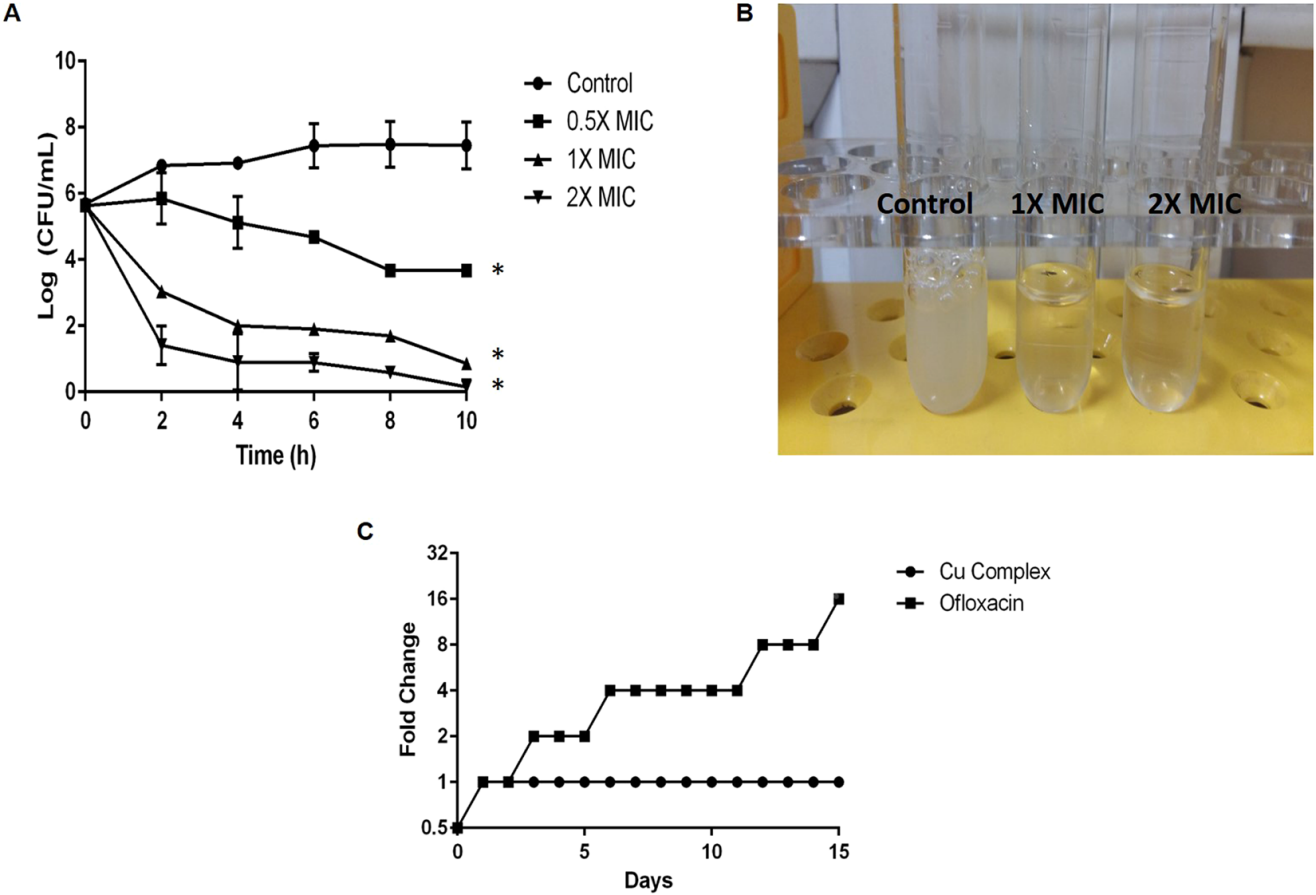
Time-dependent killing kinetics of bacteria by Cu complex. (**A**) *S. aureus* cells were treated with different concentrations of Cu complex (0.5 × MIC, 1 × MIC, 2 × MIC) and colony forming unit (CFU/mL) was measured every 2 h for a period of 10 h. Data represent mean ± SD of three independent experiments (*p < 0.05 compared to untreated control). (**B**) Cu Complex treatment resulted in complete cell lysis. (**C**) Serial passaging of *S. aureus* with sub-inhibitory concentrations of Cu complex and ofloxacin. Data is representative of two independent experiments.

We have determined the MICs for MRSA and MSSA clinical isolates of *S. aureus* obtained from human (n = 20) and animal infections (n = 20). The MSSA isolates from animal, and human infections exhibited a MIC of 6.25 μg/mL whereas for MRSA the MIC ranged from 6.25 to 12.5 μg/mL. The time-kill kinetics of Cu complex at different concentrations, 0.5 × MIC, 1 × MIC and 2 × MIC was monitored (Fig. 3A). In MHB medium inoculated with 10^6^ CFU/mL of *S. aureus* ATCC 29213, the bacterial growth curves showed the inhibitory effect of Cu complex on this strain. Bacterial growth was completely inhibited under Cu Complex concentration of 1 × MIC or 2 × MIC for 2–10 h. Complete cell lysis can be observed at 1 × MIC and 2 × MIC (Fig. 3B).

No resistance against Cu complex was observed during continuous serial passaging with sub-inhibitory concentration for 15 days (Fig. 3C). However, resistance was developed against ofloxacin within few days of exposure. We did not find any mutant lines of *S. aureus* resistant to Cu complex, plated on MHA plates containing a variable concentration of Cu complex (2 × MIC, 4 × MIC & 10 × MIC).

### Anti-biofilm activity

**Figure 4:**
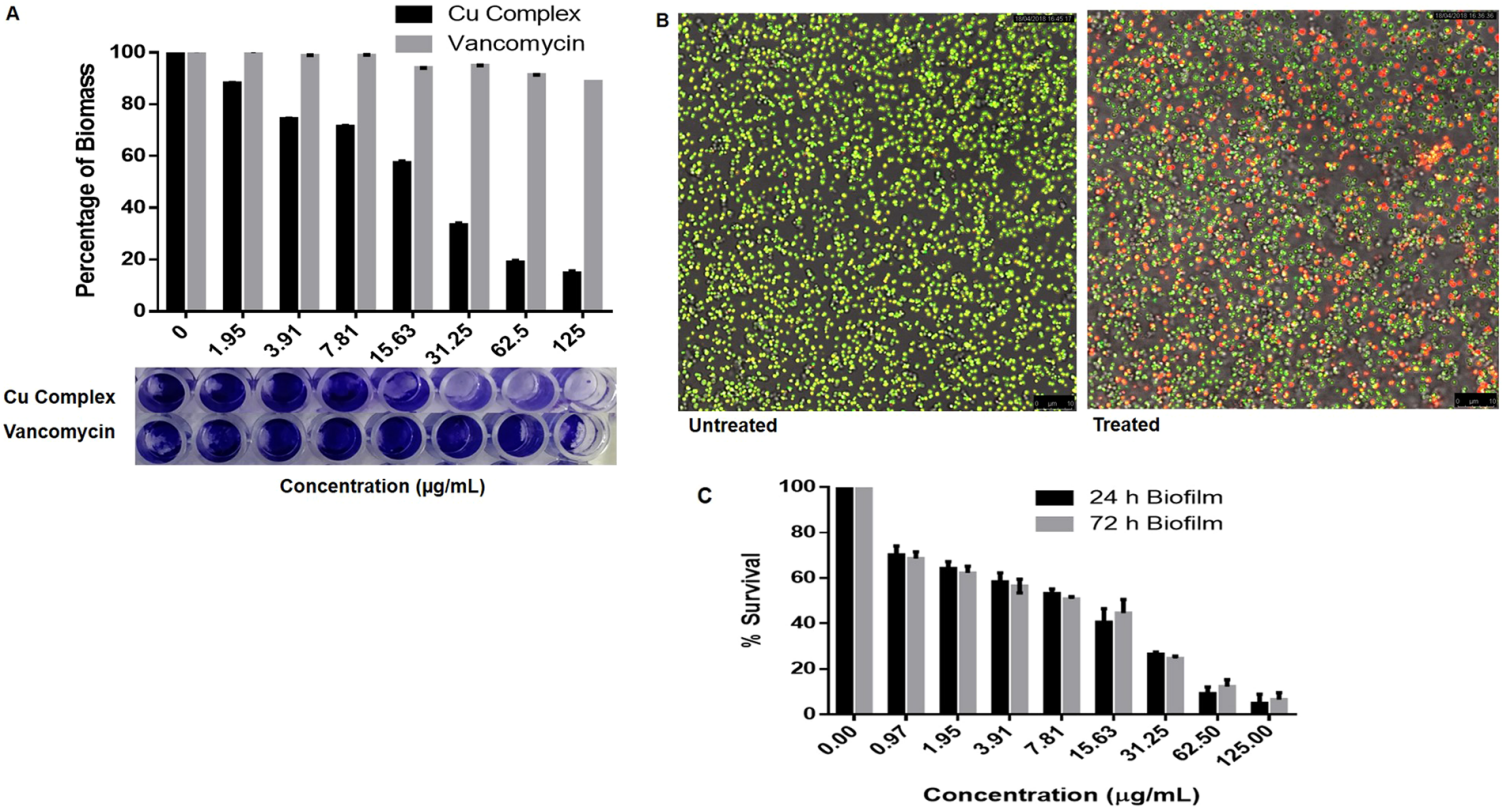
Antibiofilm activity of Cu complex. (**A**) The effect of different concentration of Cu Complex and vancomycin on 24 h old biofilms of *S. aureus* was determined by Crystal violet (CV) assay. The biofilm was stained by crystal violet and imaged using digital camera, after that the dye was dissolved in 33% acetic acid and absorbance was measured at 595 nm and presented as percentage of biofilm compared to untreated wells “control”. All experiments were done in triplicate, the bar represent mean ± SD. (**B**) Confocal images of the biofilm treated and untreated with Cu complex observed using SYTO-9 (live cells) and PI (dead cells) dye. (**C**) The inhibitory effect of different concentrations of Cu complex on 24 h and 72 h old biofilm measured using resazurin assay. Data represent mean ± SD of three independent experiments, p > 0.05.

Cu complex was significantly effective in disrupting the 24 h pre-formed biofilm and killing the bacterial cells in comparison with vancomycin (p < 0.05) which was revealed by a reduction in biomass percentage (Fig. 4A). Confocal imaging using SYTO-9 and PI showed the killing of biofilm cells treated with Cu complex in comparison to control (Fig. 4B). The resazurin viability assay showed 50% inhibition (Mean IC_50_) of 24 h and 72 h old biofilms at 9.18 ± 0.16 μg/mL and 8.51 ± 0.68 μg/mL of Cu complex, respectively (Fig. 4C). There was no significant difference (p > 0.05) between Cu complex activity to kill 24 h or 72 h old biofilm.

### Cell membrane permeability

**Figure 5:**
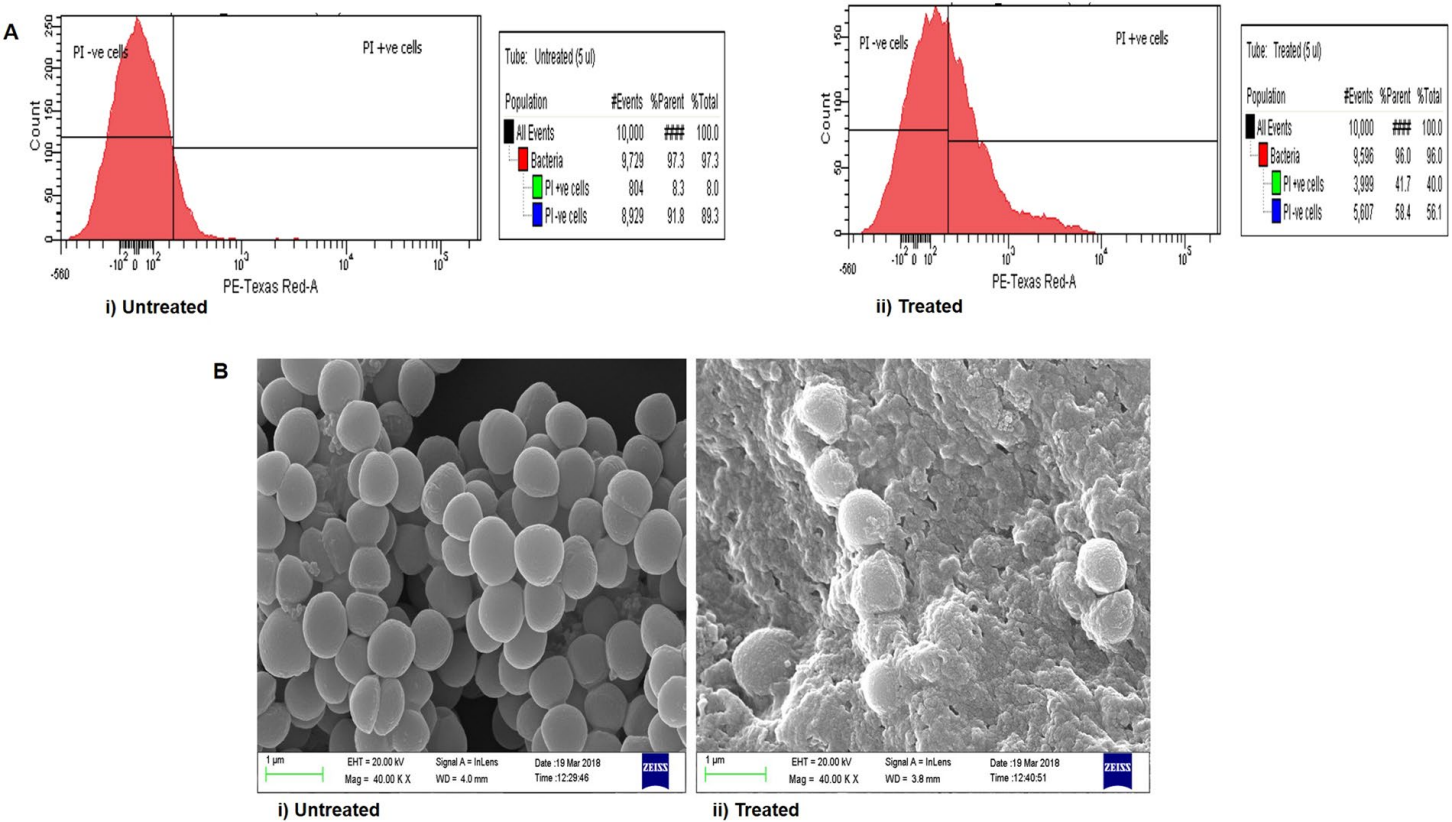
Effect of Cu complex on Cell membrane. (**A**) Cell membrane permeability was monitored by Propidium Iodide (PI) uptake using Flow cytometry: (i) Untreated cells of *S. aureus*, (ii) Cu complex (50 μg/mL) treated cells. (**B**) Scanning electron microscopy showed the effect of Cu complex on the cell membrane: (i) Untreated cells of *S. aureus*, (ii) Cu complex (50 μg/mL) treated cells.

Membrane permeability of *S. aureus* was determined by the uptake of PI using FACs. Cells treated with Cu complex and control cells with no treatment were incubated with PI and run on FACs. In treated cells, there was increased uptake of PI showing 41.7% of the cells positive for the stain as compared to 8.3% in control cells. Addition of Cu complex to *S. aureus* cells caused an increase in PI fluorescence, indicating that Cu complex permeabilizes the bacterial membrane (Fig. 5A). SEM images revealed prominent morphological alteration in the cells treated with Cu complex in comparison to control (Fig. 5B). In control, the cell membranes were found intact. However, Cu complex treated cells were showing numerous protrusion on its surface, damage to the cell membrane and cell lysis was observed.

### Intracellular Anti-*S. aureus* activity and cell cytotoxicity

**Figure 6:**
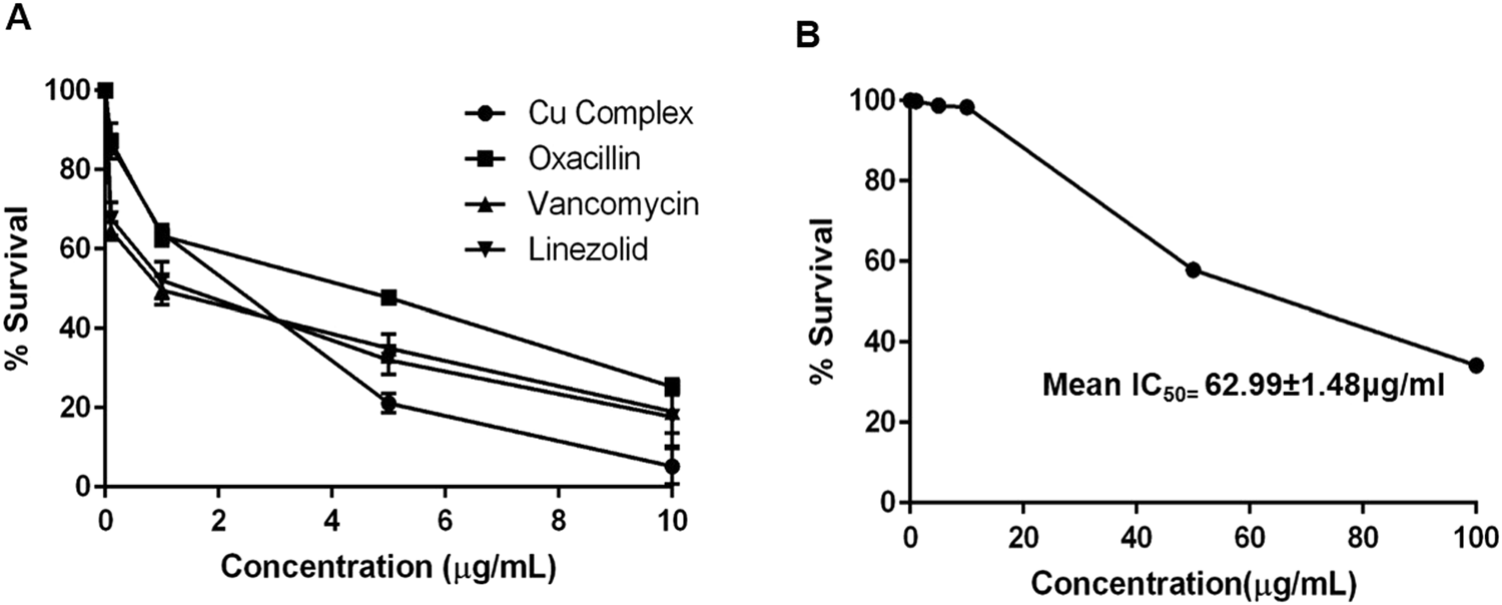
Intracellular activity of Cu complex. (**A**) Intracellular activity of Cu complex against *S. aureus* in RAW 264.7 cell lines in comparison with Oxacillin, Vancomycin and Linezolid. Different concentrations (0, 1, 5, & 10 μg/mL) were tested for all antimicrobial agents. The graph represents mean % survival of intracellular bacterial cells at different concentrations for each drug. All experiments were done thrice. (**B**) Cell cytotoxicity of Cu complex was determined using RAW 264.7 cell line. Cells were treated with different concentrations of Cu complex ranging from 0 to 100 μg/mL. The graph represents mean % survival at different concentrations. All experiments were done thrice.

We also assessed the antibacterial activity of Cu complex against intracellular *S. aureus* in RAW 264.7 cell line and compared its efficacy with different antibiotics (Oxacillin, Vancomycin, and Linezolid). Cu Complex showed a maximum bacterial reduction of 96.9% at a concentration of 10 μg/mL; however, none of the other antibiotics had shown a reduction in bacterial load of more than 77.3%. We have also observed bacterial clearance activity at 0, 0.1, 1, 5 and 10 μg/mL of Cu complex and compared with other antibiotics (Fig. 6A). Cu complex cytotoxicity was assessed using RAW 264.7 cells at variable concentrations ranging from 0 μg/mL to 100 μg/mL for 24 h. The inhibitory concentration at which 50% toxicity of RAW 264.7 cells occurred was 62.99 ± 1.48 μg/mL (Fig. 6B).

## Discussion

The ability of the bacterial pathogens to develop resistance against major classes of antibiotics has placed human and animal life at risk. *S. aureus* is one such pathogen, which infects broad host range and causes mild to chronic infections^13,32–34^. The additional worrisome factor associated with *S. aureus* infection is their ability to form a biofilm, which makes them highly resistant to antimicrobials and host attack^3,13,35,36^. There is an urgent need for antibiotics, which can not only kill planktonic cells but can also eradicate biofilm. In light of the current status, preliminary studies with Cu complex showed potent antibacterial and anticancer activity as reported previously, which encouraged us to explore its activity further^22^. In this study, we have evaluated Cu complex efficacy against different forms of *S. aureus* infections: planktonic cells, biofilm cells, and intracellular cells.

Cu complex showed increased susceptibility against standard isolates of *S. aureus* ATCC 29213, ATCC 33592 and ATCC 700699. We have also determined the efficacy of Cu complex in clinical isolates of *S. aureus* from human (MRSA = 10, MSSA = 10) and animal (MRSA = 10, MSSA = 10) infections. The planktonic growth of all isolates was inhibited and showed a MIC ranged from 6.25 to 12.5 μg/mL.

*S. aureus* isolates are notorious for developing resistance against various classes of antibiotics^37^. Therefore, to be a valid treatment option, it is crucial to determine the ability of *S. aureus* to develop resistance against Cu complex. We were unable to find mutant of *S. aureus* resistant to Cu complex when plated on media containing the compound (4 × MIC and 10 × MIC). Additionally, serial passage of *S. aureus* for 15 days in the presence of Cu complex also failed to detect resistant mutant. These results highlight the potential of Cu complex as a treatment option.

Biofilm infections are of much bigger concern in comparison to infections caused by planktonic cells^1,8–13,28^. In case of biofilm-associated infections, higher concentrations of drugs are required, which makes such infections extremely difficult to cure^1,7,10,13,28^. The Cu complex was also able to kill bacterial cells in 24 and 72 h pre-formed biofilms with no significant difference, which enhances its probability to be an effective antimicrobial agent. These results emphasise that Cu complex could be developed as antimicrobial agents for treating planktonic and biofilm-associated infections.

Further, *S. aureus* can invade and survive inside the host cells causing chronic infections. At the intracellular stage, the treatment becomes very challenging due to the difficulty of antibiotics passage through the cellular membranes. Therefore, in addition to extracellular antimicrobial activity, intracellular antimicrobial activity is also required. We evaluated the intracellular antimicrobial activity of Cu complex. We found that Cu complex was able to reduce the bacterial load to more than 95% at an intracellular level as compared to Vancomycin (75.67%), Linezolid (82.34%), and Oxacillin (74.67%) at 10 μg/mL concentration of each. Cell cytotoxicity analyses revealed 50% toxicity observed at 62.99 ± 1.48 μg/mL. However, this value is quite higher than the concentration at which MRSA growth can be inhibited. Owing to the cytotoxicity caused by Cu complex we are also trying to modify the complex to reduce its cell toxic effect.

Overall, the Cu complex exhibit substantial antimicrobial activity against both MRSA and MSSA clinical isolates of human and animal origin. It is not only effective in killing planktonic cells but also has antimicrobial activity against biofilm and intracellular bacterial cells. Thus, these studies warrant further in-depth investigation for the development of Cu complex as an effective agent against biofilm-associated and chronic intracellular infections.
